# “You Have to Set the Story You Know Aside”: Constructions of Youth, Adulthood and Senescence in *Cinderella Is Dead*

**DOI:** 10.3390/h11010025

**Published:** 2022-02-10

**Authors:** Michelle Anya Anjirbag, Vanessa Joosen

**Affiliations:** Department of Literature, University of Antwerp, 2000 Antwerp, Belgium; michelle.anjirbag-reeve@uantwerpen.be

**Keywords:** fairytales, fairytale rewritings, age, gender, age studies, young adult literature, fantasy, social criticism

## Abstract

As with other twenty-first-century rewritings of fairytales, *Cinderella is Dead* by Kalynn Bayron complicates the classic ‘Cinderella’ fairytale narrative popularized by Charles Perrault and the Brothers Grimm for new audiences, queering and race-bending the tale in its decidedly feminist revision of the story. However, as we argue here, the novel also provides an interesting intervention in the construction of age as related to gender for its female protagonists. Drawing on Sylvia Henneberg’s examination of ageist stereotypes in fairytale classics and Susan Pickard’s construction of the figure of the hag, we explore the dialogic between the fairytale revision, traditional fairytale age ideology and the intersection of age and gender in this reinvention of the classic narrative. By focusing on constructions of age, particularly senescence, we demonstrate how complex constructions of older characters might aid in overall depictions of intergenerational relationships, and how these intergenerational relationships in turn reflect historical and cultural impetuses of retelling fairytale narratives.

## 1. Introduction

While fairytales have historically given voice to concerns that occupied their tellers in the past (Zipes 2006; Tatar 1999; Warner 1995), fairytale rewritings continue this tradition of confronting the real through stories of magic and wonder. As Cristina Bacchilega argues, “Fairytales interpellate us as consumers and producers of transformation” (Bacchilega 2013, p. 3), and can, thus, stimulate societal reflection and change. The most powerful rewritings not only shed new light on the traditional fairytales, but also make them relevant in addressing contemporary cultural trends and societal issues. Most recently, Kalynn Bayron’s 2020 young adult novel *Cinderella is Dead* has been lauded for its race-bending, queering and feminist revision of the fairytale made famous by Charles Perrault and the Brothers Grimm. In this article, we focus on an additional aspect of the novel—its construction of age. 

For our discussion, we draw inspiration from analyses of age in fairytale classics (a.o. Schmiesing 2014; Murai 2018). Sylvia Henneberg’s categorization of ageist stereotypes in particular provides a useful starting point to examine how *Cinderella is Dead* engages with the traditional fairytale’s age ideology. However, this discussion of fictional age constructions also has wider relevance, given that fairytales’ depiction of old age has become a template for viewing older women in reality more broadly. In her 2016 handbook *Age Studies: A Sociological Examination of How We Age and Are Aged Through the Life Course*, Susan Pickard uses the figure of ‘the hag’ as a red thread to explore discourses on the life course; the hag “epitomizes our view of ageing as decline and loss of self and our horror at the loss of choice and control that finds its ultimate expression in death” (Pickard 2016, p. 23). Therefore, literary reconfigurations of the hag figure not only provide intertextual revisions of the fairytale, but may also be related to the wider implications of this figure and related discourses on age in reality. Such reflections are timely, especially in the light of the rising old-age ratio in the Western world and public debates where the interests of younger and older generations are pitted against each other, such as climate change and the COVID-19 lockdowns. Since age is always a relational concept, we will not only focus on senescence in this article, but also on the construction of youth and adulthood in *Cinderella is Dead*, as well as intergenerational relationships.

In the kingdom of Mersailles and the town of Lille, where Bayron’s story is set, young girls have to live by a set of rules that is derived from the ‘Cinderella’ fairytale. Once they turn sixteen, they are obliged to attend a ball organized by the king, so that they can be chosen by the men who visit the ball to pick a wife. Sophia, the protagonist, is in love with her friend Erin. While reluctant to go to the ball from the start, she is horrified when seeing Liv, another friend, being degraded there, only to find out later that she has been killed. Sophia makes an escape from the ball and is determined to bring down the king and his violent patriarchal system. While fleeing, she receives help from a young woman named Constance, who teaches her that the ‘Cinderella’ tale that the girls of Lille have been fed is a distortion of history. The real Cinderella did not fall victim to her evil stepmother and wicked stepsisters; instead, they were allies in confronting the oppressive Prince Charming after he had killed her parents. As a love story unfolds between Sophia and Constance, they find refuge with the fairytale godmother, who lives in a cottage in the forest. The girls learn that Prince Charming has been able to rejuvenate himself and to return to Lille under different guises, so that he has been able to rule over the town for over two centuries. Together the three women devise a plan to defeat the King and liberate the people of Mersailles. 

Without exception, the main characters in *Cinderella is Dead* are all described as having brown skin. As a title written by an author of color, the book has been praised for representing #Ownvoices, and it confronts readers with how their mental imagery may have been shaped by the whiteness that prevails in most fairytale books (with some exceptions, e.g., Robert San Souci and Brian Pinkney’s *Cendrillon: A Carribean Cinderella*). Bayron’s novel resembles popular narratives of the 21st century, such as the Netflix adaptation of Margaret Atwood’s *The Handmaid’s Tale* and Suzanne Collins’ *The Hunger Games*, in using dystopic fantasy to address the commodification and oppression of young women in real life. By exposing the heteronormative structure of ‘Cinderella’ and replacing it with a love story between two young women, *Cinderella is Dead* also succeeds in queering the traditional fairytale on various levels.

Classic fairytales have been criticized for imparting dated worldviews when it comes to race, gender and sexuality. In her 2010 article *Moms Do Badly, but Grandmas Do Worse*, Sylvia Henneberg argues that in the best-known fairytales, sexism intersects with ageism, resulting in stereotypes about older women that are hard to shed. They are typically given the part of “the wicked old witch, the selfless godmother, or the demented hag” (Henneberg 2010, p. 128). In ‘Cinderella’ specifically, Henneberg points at the ‘dead mother plot’ that results in the absence of a nurturing mother figure. The fairy godmother who appears in Perrault’s version of the tale is an example of a positive ageist stereotype. She is attributed good traits, such as magical powers and kindness, but still raises concern because her characteristics remind Henneberg of the “wise old elder” figure. She explains that “(o)ne big problem with many of these benevolent figures is the kind of advice they give”, in particular when they foster female compliance, while “another problem is the fact that these elders give advice from an aloof position, far removed from the center of action” (p. 129). The only purpose of the fairy godmother and similar older figures in fairytales is to assist the young in their pursuit of happiness. For this reason, Henneberg calls them “self-sacrificial lambs in disguise. At first sight, they seem to be important leaders, but upon consideration they are relegated to the margins, existing only to develop other characters and plot lines rather than their own” (p. 129). Referring to research on the intersection of ageism and sexism by MacDonald and Rich (1983/1993), Henneberg points at the risk that this position holds: “The female elder is tolerated if she has the will and ability to boost, support, guide and serve the young”, only to become invisible again once she has fulfilled that part (p. 129).

*Cinderella is Dead* substantially extends the role of the fairytale godmother, who also receives a name in the book—Amina. She appears as a mysterious and ambivalent figure who is crucial to the development of the plot and the creation of narrative suspense. Moreover, the novel also expands the interaction between the fairy godmother and the adolescent protagonist of the story. In what follows, we offer a close reading of passages from the novel to explore the age ideology that *Cinderella is Dead* creates. As a fairytale rewriting that thematizes the transmission of knowledge and stories, *Cinderella is Dead* is heavily invested in a metafictional play that questions how stories can reflect authors’ and shape readers’ worldviews. Samantha Stephens and Leonie Rutherford argue that metafictional texts for young readers “can help to renegotiate limiting discourses of childhood” because they show how narratives by adults can be deconstructed, creating the possibility of more agency for the young. The risk is that limiting age norms can be reinforced in this process, Stephens and Rutherford write, since such metafictional novels “can simultaneously function to reinforce generalizations of adulthood that foreground intergenerational conflict and other ageist sentiment, thus limiting the subversive potential of these texts” (Stephens and Rutherford 2021, p. 58). While Bayron’s novel is heavily invested in exposing and rewriting traditional fairytale roles, as well as in creating a suspenseful plot, its construction of age may fall somewhat under readers’ and critics’ radars, especially since critical examinations of age are far less prevalent in popular and academic discourse than reflections on norms relating to gender, race and sexuality. Stephens and Rutherford’s observation begs the question if and how *Cinderella is Dead* rewrites the roles of the fairytale godmother and other adult figures in ‘Cinderella’ and what the effect is on the novel’s age ideology and potential contribution to intergenerational understanding. Before we zoom in on the adult figures, however, we first focus on the novel’s metafictional features and the age norms it addresses for youth.

## 2. “Don’t Question the Story”: Youth and Infantilized Womanhood in Mersailles

Fairytale scholars such as Jack Zipes have shown that the literary fairytale has potential for both didacticism and subversion, and as such may serve as a cultural socialization tool. Zipes notes that:

Educated writers purposely appropriated the oral folktale and converted it into a type of literary discourse about mores, values and manners so that children and adults would become civilized according to the social code of that time. By the eighteenth century, the writers of fairytales for children such as Sarah Fielding and Madame Leprince de Beaumont *acted* ideologically by presenting their notions regarding social conditions and conflicts, and they *interacted* with each other and with past writers and storytellers of folklore in a public sphere.

(Zipes 2006, p. 3; emphasis original)

Two competing ‘Cinderella’ tales are embedded within the narrative of *Cinderella is Dead*: a “palace-authorized text” and a version of the story that is kept by Constance and her family. By thematizing the differences between the two versions and their functions, the novel highlights the interactions between society and fairytales, the role of a fairytale narrative as a “civilizing” tool across age groups and the genre’s potential for reinforcing as well as challenging authority. The palace-approved ‘Cinderella’ combines elements from Perrault’s version (the fairytale godmother) and the Brothers Grimm’s (the mutilation of the evil stepsisters). It is used as an overt tool for establishing and maintaining patriarchal norms. With its depiction of Cinderella’s modest beauty as the feminine ideal, the palace-authorized fairytale “showcase(es) ‘women’ and mak(es) them disappear at the same time…(transforming them) into man-made constructs of ‘woman’” (Bacchilega 1997, p. 9). Girls in Mersailles are expected to learn the tale by heart from a young age and aspire to be as good and pure as Cinderella in order to secure themselves a good future. They are taught to fear being the same as the stepsisters (Bayron 2020, p. 18). As such, the story is used in the novel to perform a function that has also been ascribed to fairytales in reality, albeit in a more overt way than usual—to “regulate and limit the nature of children’s development and regulate the sexual relations and social comportment of young adults” (Zipes 2006, p. 32). The palace-approved Cinderella text exemplifies the fairytale’s potential “to indoctrinate children so that they will conform to dominant social standards that are not necessarily established on their behalf” (Zipes 2006, p. 34). In Mersailles, Cinderella’s story is not just told as a tale of wonder, but also an account of history and as a manual for girls on how to prepare for being chosen by a man.

In an early feminist critique, Lieberman ([1972] 1989) writes that “(m)arriage is the fulcrum and major event” (p. 189) of fairytales such as ‘Cinderella’, which typically end with the wedding but do not show what happens after that (p. 199). *Cinderella is Dead* sketches a bleak picture of married life for women. The country of Mersailles as seen through Sophia’s eyes is a brutal place. She recounts scenes of domestic abuse, homophobia and LGBTQ suppression, and misogyny as a not only normal but expected part of life. To avoid such fates, girls are instructed to “put their faith in the story” (Bayron 2020, p. 21). Those who question it or provide alternative narratives are punished or put to death. While men are also affected by the situation, the deployment of the fairytale narrative as a tool of socialization, social coercion and control leads to an interesting interrogation of the intersection of age and gender, where age norms within the narrative become a force multiplier upon the gender norms that restrict women’s lives in Mersailles.

Bayron’s novel simultaneously amplifies and thematizes tendencies that age scholars have identified in Western society, particularly the cult of youth and the decline narrative of old age. Pickard (2016, pp. 3–5) points out that an age ideology that constructs the process of growing older as a narrative of decline is particularly harmful to women, who are glorified but also infantilized in youth and vilified in adulthood. In *Cinderella is Dead*, the gender norms promoted by the palace-authorized version of Cinderella’s story are heavily intertwined with age norms. The annual ball that reenacts the events of Cinderella’s ball night is mandatory for women once they turn sixteen. Those who are not chosen by a husband for three balls in a row are considered forfeit to the crown (Bayron 2020, p. 11). A girl’s sixteenth birthday is explicitly marked as the moment she comes of age into her value in society, but this is not a process in which she gains agency. Her value is tied to her youth and the reputation of her family. At best, her father will have paid a suitable husband to pick her; at worst, she is at the mercy of any man who chooses her. As such, sixteen-year-old girls are consumable objects; those who do not manage to bring much or present themselves as a ‘prize’ become forfeit. In fact, as readers learn during the story, some of those girls are literally consumed by the king so that he can extend his life and restore his youth. 

As with other narratives, such as Disney’s *Tangled*, *Cinderella is Dead* combines a fairytale narrative with a quest for youth trope. As Sanna Lehtonen points out, “magic age-shifting occurs as a motif in folk literature” (Lehtonen 2013, p. 42) and is also popular in fantasy and horror stories. One of the truths revealed about the “real” story of Cinderella is that King Manford is Prince Charming, and every other one of Mersailles’ rulers. Having been brought back to life by his mother before he took the crown as Mersailles’ savior, he has been artificially extending his resurrected existence for 200 years by feeding vampirically off of the life force of his citizens, especially those who rebel against him or are otherwise forfeited to the palace, including Cinderella herself. Those whose life force Manford feeds off of to maintain his existence are visibly aged beyond their years (Bayron 2020, pp. 33, 95–96, 131, 271, 339–340, 349–356), while he is rejuvenated. Consider the description of Sophia’s friend Liv when she is found dead:

Liv’s hair, once brown, is now white as snow. Her skin is shriveled and ashen gray. Her arms are drawn up in front of her, her hands rigid, fingers curled into claws.

The description of seventeen-year-old Liv’s body is reminiscent of the hag that Susan Pickard sketches, with “crow’s feet and wrinkles…brittle hair…greying skin” (Pickard 2016, p. 3). As Lehtonen argues, “age-shifting often functions as a reward or punishment motif in tales” (Lehtonen 2013, p. 43). In Mersailles, this rapid ageing into death is the ultimate punishment for girls who do not comply with the rules of the ‘Cinderella’ tale. The value of young women is bodily and they are quite literally the king’s ‘fountain of life’, a reflection of the genre-spanning literary trope of the search for eternal youth and immortality. 

Age norms apply differently to men and women in Mersailles, illustrating Pickard’s observation that “whilst non-adult stages are particularly disadvantaged, ‘adulthood’ is at once chronologically and symbolically constituted, meaning that some members of this category are more ‘adult’ than others” (Pickard 2016, p. 3). After they have turned sixteen, women only have three chances to be chosen at the ball. Those not married by eighteen are considered spinsters (pp. 33–34). There are no similar requirements for men, who are at no point compelled to attend (Bayron 2020, p. 34). Men can go to the ball at whatever age they want. In Mersailles, women of all ages live under curfew and are controlled by a legally designated male head of household. They do not own property but are owned themselves. While other men do not wield the same vampiric power as the king, they certainly treat young women as a commodity for consumption and use, with some trying to claim “two girls at a time” (Bayron 2020, p. 126) and others planning to get rid of their wives to replace them with a younger one. Notably, Cinderella also dies at age 38 (Bayron 2020, p. 271), never reaching old age, or even a true middle age, although her body appears old due to Manford’s abuse. The central figure of the narrative that is meant to shape the lives of women in Mersailles does not achieve maturity or have a future; her death makes it impossible to imagine a woman’s value beyond that granted by her youth. This becomes painfully clear in the figure of the seamstress who has made Sophia’s dress. Not only is the seamstress already subject to domestic abuse and economic exploitation by her husband, she is scapegoated after Sophia’s rebellion at the ball. The seamstress is accused of helping Sophia and decapitated. Abjected from society, only in the face of death is she granted some agency when she is able to express her despise of the king.

Women are, thus, not only consumable and abjectable, but have a short shelf life, which also adds to the amount of control exerted over women through the manipulation of their status in the kingdom through their age. While the “dynamics of emplotment” are pertinent to the social practices of Mersailles, where the Cinderella narrative “has been hegemonically utilized to emplot or frame…lives within a heteronormative capitalist economy” (Bacchilega 2013, p. 6), these dynamics are weighted unequally on gendered lines, which cannot be disentangled either from the implicit age dynamics of the palace-authorized Cinderella narrative or the reality of her death at age 38. During her entire marriage, she was held prisoner by Prince Charming and deprived of the agency and responsibilities associated with adulthood. If Pickard argues that “some members of this category are more ‘adult’ than others” (p. 3), then Cinderella was constituted as ‘less’ adult, as are the women who are expected to tread in her footsteps.

## 3. Adulthood and Agency in Mersailles: The Other ‘Cinderella’ Story

The version of the Cinderella story that Constance knows, however, both in its oral form and through an illustrated book, acts as a counternarrative to the approved story. As Sophia and Constance uncover the full tale and history, the palimpsestic narratives of Cinderella within the novel and framing the novel demonstrate “how the narrative construction and manipulation of the tale of magic contribute to making different ideological effects possible within specific historical and social contexts” (Bacchilega 1997, p. 8). “You have to set the story you know aside”, Constance tells Sophia, as she gradually shares the details of the alternative Cinderella tale. This narrative gives Sophia glimpses of adult female agency and bonding both in the content of the story and in the way that it has been shared. Knowledge passed down through women, recorded by women and preserved by women in the written, illustrated and oral forms demonstrates gendered intergenerational narrative transmission of story as a subversive force, echoing the efforts of female fairytale writers of the seventeenth and eighteenth centuries in France who “sought to subvert the male code and replace it with a more liberal one favorable to the predilections of educated women, who wanted more power to determine their lives” (Zipes 2006, p. 32). As Marina Warner notes, the “fairytale offers a case where the very contempt for women opened an opportunity for them to exercise their wit and communicate their ideas”, especially around the norms of women’s lived experience (Warner xix), an observation that takes as given intergenerational exchange undertaken with a degree of discretion, where adult and older women are integral to the survival of the younger generation, and information and experience are not necessarily passed on overtly. This is no less the case in Bayron’s Mersailles. In Constance’s observation, “the palace underestimates the resourcefulness of women forced into a dark and dangerous place” (Bayron 2020, p. 162). Indeed, intergenerational networks of information-sharing and rebellion are what in the end allow Sophia and Constance to bring down Manford.

From the oral history passed down in her family, Constance knows that Cinderella bonded with her stepmother and stepsisters: “Wicked? No. Stepsister, yes” (Bayron 2020, p. 140). Moreover, as Constance points out about Cinderella’s mother, “In the story, she doesn’t even have a name” (Bayron 2020, p. 157). In the alternative tale, by contrast, all the central female figures are named, which is symbolic of them being recognized as individual personalities rather than as stereotypical antagonists in a story. Constance shares that the stepsisters lived on after Cinderella was killed, had children and that the generations coming after them kept the memory of their rebellion against Prince Charming alive. In doing so, they were aided by letters that were passed down the generations and a fairytale book. When Karen Rowe argues that telling fairytales is “semiotically a female art” (Rowe [1986] 2014, p. 16), she does not only refer to text, but also to images. Rowe recounts Ovid’s tale of Philomela, who told about her rape by weaving it into a tapestry after her tongue was torn out. In the book that is passed down in Constance’s family, the ‘Cinderella’ tale is illustrated with slightly different pictures than the palace-approved version. The pictures hint at female intergenerational bonding between Cinderella and her stepmother Lady Davis, whereas the palace-approved edition of the same book stresses female rivalry: “In Constance’s version, Lady Davis is leaning forward, her hand extended, her face gentle, her eyes full of sorrow, and Cinderella isn’t bowing as much as she is kneeling, like she’s just collapsed” (Bayron 2020, p. 292). As Sophia and Constance understand, the book shows how Cinderella is supported by her stepfamily after her father has been killed. The alternative fairytale book, thus, offers the nurturing adult female that Henneberg was missing in fairytales with a ‘dead mother plot’. Moreover, in the apocryphal history that Constance’s book offers, Lady Davis is not a magical figure but offers a kind of support that is also available to readers in real life. 

The comparison between the two fairytale books further reveals a problematic fascination with violence against female bodies in the palace-approved ‘Cinderella’, which follows the Brothers Grimm’s tale; the stepsisters cut off their heels and toes to fit into the glass slipper, and at the end of the story, their eyes are pecked out “while their mother was forced to watch...In Constance’s book, they are exiled without all the gory details” (Bayron 2020, p. 292). In drawing this parallel between the palace-approved version of ‘Cinderella’ and the Brothers Grimm’s canonized tale, *Cinderella is Dead* reveals that aspects of Mersailles’s misogyny, in particular the fascination with violence against female bodies, also apply to the real world. To some extent, the novel draws on that fascination itself to create suspense, in its depiction of the drained victims of Manford and the gruesome beheading of the seamstress. However, whereas in the Grimm’s tale this kind of violence is simply accepted as a form of just punishment, in *Cinderella is Dead* it gives the characters a reason to form alliances and resist.

## 4. Old Age

Older women are included in this network of female rebellion and truth-telling. *Cinderella is Dead* does not just have a ‘dead mother plot’ but also a ‘dead grandmother plot’. In the margins of the story, Sophia’s grandmother is celebrated for her revolutionary spirit. The old woman would not be fooled by superstition, shared secret information with friends about Cinderella’s tomb and actively rebelled against the king:

My grandmother was like a storm, wild and unpredictable and sometimes a little too harsh for my father’s comfort. When she would speak about the ball, she never made it seem like it was something that was inevitable. She always used the word if when she spoke of it. If the day came for Sophia to go to the ball. If we were still doing this when young Sophia got older. It was her spitfire spirit, her hatred of the way Lille was run that got her killed. She had said too much to the wrong person, and the palace guards came to get her on a cold rainy afternoon. She kicked one of them on the way out the door. A week later my father received a letter that informed him where he could pick up her body for burial.

(Bayron 2020, p. 71)

Sophia is positioned as her grandmother’s heir, with her parents trying to mitigate her defiant spirit for fear that she might end up killed as well. Sophia’s memory of her grandmother becomes a touchpoint of real rebellion from a woman she knew, a woman who had a lifetime of experience in a society that was becoming more oppressive to women and chose to fight anyways. As such, her grandmother becomes an important grounding factor for Sophia as she enters into a world where fairytale figures from Cinderella to the stepsisters to the fairy godmother become far more real than the story Sophia thought she knew.

In Marina Warner’s account of female empowerment through telling fairytales, however, one figure who is integral to the ‘Cinderella’ tale takes prime place—the fairy godmother. Through this character, Warner argues, as well as through the figures of stepmother and witch, “the older generation speaks to the younger in the fairytale” and tries to win their favor and loyalty, whether it is by threatening them with evil or rewarding them with good fortune (Warner 1995, p. 227). The godmother does the latter—she is the older guardian, the “wonder-worker” (Warner 1995, p. 227) who helps Cinderella to secure a good future. When an equivalent of the fairy godmother pops up with the figure of Amina, readers might expect that she will be crucial in assisting Constance and Sophia. The girls find refuge in her house in the woods and at first Amina fulfills the promise of the older female helper by sharing knowledge and letting the girls share in some of her magic. Through Amina, the girls learn about Manford’s vampirism, and she assists them in plotting their attack on Manford. However, the ominous cottage in the woods that she dwells in, her independent spirit and her knowledge of dark magic also link her intertextually to another fairytale figure—the witch. She takes an outsider position and appears as an enigma to the girls, with her reclusive life and incredible old age; it is soon established that Amina is over two centuries old. She thus functions as a plot device to create suspense—can this older woman with magical powers be trusted or not? 

Whereas the fairy godmother in ‘Cinderella’ secures the continuation of the patriarchal status quo, the witch may in fact be a more suited figure to assist Sophia and Constance in their challenge of authority. As a witch figure, Amina performs what Kay Turner refers to as “a potent blend of defiance and transgression that rattles the nerves of the normative” (Turner 2019, p. 29). David Punter writes:

Just as fairytales speak to imaginings of hope, promise and fear in terms of the putatively adult world...so the witch occupies this liminal space where many things might be possible, many things are forbidden and forbidden, transgressive knowledge is the ambition, the hope of circumventing the world of the normal and the everyday.

(Punter 2017, p. 68)

Sophia and Constance can be argued to be partly trained as witches when Amina shows them how they can see into their own futures by bathing in her pond and teaches them necromancy to bring the deceased Cinderella back to life. However, a sense of danger is attached to this figure, which cannot be shed. Aspects of Amina are reminiscent of their arch enemy King Manford—her distrust of Gabriele, one of the stepsisters, as well as her unnaturally long life. While Manford has to feed off other bodies to rejuvenate himself, Amina seems to have sustained life through a kind of self-sufficient stretching of vitality. She admits that she had a crucial role in rejuvenating him, and wants to make amends by helping Sophia and Constance. Once they have put their trust in her, however, she also betrays them. 

In a climactic fight at the end of the book, it turns out that Amina is Prince Charming’s mother—a move that is reminiscent of Dreamworks’ *Shrek 2* (2004), where the two figures of the fairy godmother and Prince Charming are similarly related and cast in the roles of antagonists. In *Cinderella is Dead*, Amina raised her son from the dead, and once they found out that he could extend his life by feeding on a beggar woman, she let him go his way. While Sophia and Constance believed that Amina was helping them to get into the palace to defeat the king, it is revealed that she promised him to “deliver” the girls to him. But even that may be another ruse. One might wonder why Amina shared so much of her life story with Sophia and Constance, even if she withheld the crucial fact that she is Prince Charming’s mother. Was it because she is a skilled liar who knows that her ruse will be more convincing if it is partly based on truthful facts? Or did she feel a genuine need to share her personal history with the two younger women? As Sophia reminds Amina what she has done to the people of Mersailles, she starts crying: “I can’t stop the tears running down my face. I thought you cared about me. How could you do this?” (Bayron 2020, p. 365). What happens next is a scene that is loaded with ambivalence:

Amina draws her mouth into a straight line as she approaches me, her eyes steely. She raises Cinderella’s dagger up and gently taps the handle where the pink stone is anchored. “Just like I saw it”, says Amina. “Forgive me”. She draws a deep breath and lunges toward me.

(Bayron 2020, pp. 365–66)

As Amina attacks Sophia, she is stabbed in the back by Constance’s dagger and dies. Various aspects of her act remain puzzling until the very end. What does Amina ask forgiveness for? Her compliance in Manford’s evil acts or her attempt to kill Sophia? What did she see in the moment of divination that she refers to when she says “Just like I saw it”? Her attack on Sophia or her own death at Constance’s dagger? If Amina witnessed her own death, her assault may not be a serious attempt to kill Sophia but may rather serve to provoke Constance to stab her. If that is the case, Amina plays a crucial role in ridding the kingdom of Mersailles of his evil powers. After all, as Constance read in Amina’s book of magic: “The conjurer is bound to the raised corpse until death”. By letting herself be stabbed to death, Amina makes it possible for Sophia to kill Manford, something that was not possible while she was still alive. 

Amina, thus, appears as a particularly ambivalent figure who captures elements of the three models for older women that Henneberg criticized in the classic fairytales, while also subverting aspects of all them. On the one hand, she is an evil witch and even admits that she deserved to be burnt on the pyre: “I was only on it because the people in my village found out what I’d done. Necromancy tends to scare the faint of the heart” (Bayron 2020, p. 362). By raising her son from the dead, she created a monster, and for two centuries, she turned a blind eye to all the suffering that he caused. On the other hand, the story offers evidence that Amina does truly care for Sophia, regrets some of the evil that she has inflicted and wants to atone for it. Amina does not fit into a simplistic division of black and white characters, and her acts suggest a psychological complexity even if the reader—as with Sophia—never really knows what goes on in Amina’s mind. For this reason, it is also difficult to assess if she fits the image of the ‘ineffectual crone’. Although Constance likes to call her ‘Granny’ as a way of mocking her, Amina is only weak and unsuccessful if we take her attempt to kill Sophia seriously. If not, she has succeeded in tricking all the other characters so that Manford’s rule can be ended. With the evil acts in her past, it is obvious that Amina does not simply fit the type of the fairy godmother either, although she does share some traits with her, and could be read as what Jeana Jorgensen terms a “contesting representation” (Jorgensen 2007, p. 226) of the figure, one that is “a reaction to canonical fairy godmothers” both aiding and challenging the protagonists while “also tak(ing) on new roles in new narratives” (Jorgensen 2007, p. 217). If we accept the interpretation that Amina lets herself be killed so that Manford can die too, she could be argued to resemble “the self-sacrificing lamb in disguise” that Henneberg criticizes (Henneberg 2010, p. 129). She gives her life so that Sophia, Constance and the other women of Mersailles can live. This may have also been the reason why she has so willingly shared knowledge and magic with Sophia and Constance. However, with her troubled past, Amina does not easily fit the figure of the wise old woman, nor does she appear as a character who is only there to serve the young. She has a rich and complex backstory that is partly evoked, and partly left to the imagination until the very end. 

If we accept that Amina is atoning for her evil past in the end and chooses Sophia’s freedom over her son’s extended life, she appears as an older woman who is still capable of fundamental change in deep old age. This transformation is prompted by her interaction with the two younger women. Not only are they taught by Amina, they also confront her with the way her son feeds on young women, which is something she preferred not to think about before. While it is rare to find a so-called ‘progress narrative’—the opposite of a decline narrative—in old age (Gullette 2011, pp. 147–66), Amina possibly provides one, even if she annihilates herself in the act of saving Sophia and the other women of Mersailles.

There is no question that it is Amina’s final act that saves Sophia, Constance and the other people trapped in Manford’s cells, and through them the rest of the women of Mersailles. It is only through the destruction of Manford that a new future for the country could have been conceived of; he could only be destroyed through the unification of not only the younger and older generation, but also the unification of the truth of the past and the truth of the present. Without the intergenerational cooperation between Sophia and Constance and Amina, Amina’s willingness to eventually share her true history with Sophia and Constance, and also her willingness to listen to Sophia and Constance’s accounts of the horrors faced by the women of Mersailles, the right information to end Manford’s regime would have never come to light and the women of Mersailles could not have found a new future. As such, we read intergenerational dialogue, especially around lived experiences, as a transformative force in *Cinderella is Dead*. The dialogues between Sophia and Constance, and then with Amina, disrupt the limits placed on the potential futures for women. Through the course of the narrative there is a shift from the palace-approved Cinderella narrative’s emphasis on youth with few futures outside compliance or death for mature women, much less those who reach old age, to a world under Constance’s rule that is built on the true story of Cinderella and her family, and the intergenerational bond between women through the act of passing on stories in order to provide paths and futures for the next generation. Amina’s actions are as much a part of the realization of this new future and the liberation of the women of Mersailles, as is Sophia’s rebellion and Constance’s family’s protection of the true history of Cinderella. As such, the old mentor–young hero dynamic of many fairytales or fantasies is complicated as the actions of Sophia, Constance and Amina are interlinked, rather than Sophia and Constance simply responding or reacting to knowledge shared by Amina in the role of Henneberg’s “wise old elder” figure. Amina is a full actor with agency, both in her resurrection of Manford and entrapment of Cinderella, and her subsequent atonement and acting to change the world she had helped to create, a literal link between past, present and the future of the women of Mersailles. 

This connection between past, present and future, as well as the relationship between fantasy and lived reality, are also reflected in fairytale criticism. Notably, Warner writes:

The experiences [fairytales] recount are remembered, lived experiences of women, not fairytale concoctions from the depths of the psyche; they are rooted in the social, legal and economic history of marriage and the family, and they have all the stark actuality of the real and the power real life has to bite into the psyche and etch its design.

(Warner 1995, p. 238)

Warner, along with critics such as Zipes and Maria Tatar, has long explored fairytale history, not only in terms of the history of the genre but also how these stories are interwoven into the passing on of localized histories and cultures, with “narrative elements issue(d) from real-life experiences and customs” (Zipes 2000, p. xvii), through the mechanism of intergenerational dialogues, such as those mobilized between Sophia, Constance and Amina in the novel. As stated before, Amina’s actions are hard to read with certainty; she asks for forgiveness, although it is unclear what for exactly. What is certain, however, is that her personal history is what bridges not only the two versions of Cinderella’s history in the novel, but also the history of Mersailles as told from the authoritative construct of the palace and the more localized history passed between women of different generations, whether considering the guardianship of Cinderella’s story by Constance’s family, Sophia’s grandmother’s act of questioning the official story as her legacy to her granddaughter or even the reality of the women of Mersailles who disappear without a trace. Amina’s personal history and progress narrative, therefore, provides opportunities for change and enable growth for all generations of women in Mersailles. 

## 5. Conclusions

Recently published critical volumes further the historical approaches deployed by Warner, Zipes and others, using interdisciplinary, multimedia and social-justice-informed perspectives to look beyond specific historical and cultural contexts that established the more canonical versions of the Cinderella story to better understand how this narrative still informs multiple cultural perspectives. Suzy Woltmann’s edited collection *Woke Cinderella: Twenty-First-Century Adaptations* in particular explores “how cultural expectations have evolved” through an understanding of adaptations of Cinderella as “dialogic and heteroglossic, rather than as simple subversion” (Woltmann 2020, p. 9). Contributions to *Cinderella Across Cultures: New Directions and Interdisciplinary Perspectives*, edited by Marine Hennard Dutheil de la Rochère, Gillian Lathey and Monika Woźniak, examine the figure of Cinderella as one “multiplied to carry radically different messages and serve competing interests and values”, where she is both a “reified stereotype” and “an emblematic fairytale heroine” or “embodiment of female resilience and resistance” (Hennard Dutheil de la Rochère et al. 2016, p. 1). Similarly, Christy Williams’ *Mapping Fairytale Space: Pastiche and Metafiction in Borderless Tales* provides insights into how “fairytales help the characters and audiences navigate the present…(becoming) a way of thinking through problems and solutions, of understanding what no longer works in order to forge ahead…a way of imagining the real world otherwise” (Williams 2021, p. 4). Bayron’s novel, especially in the way it emphasizes intergenerational discourses, complicates how age ideology underpins the wonder of the tale that readers might think that they know, where a magical benevolent fairy godmother swoops in to solve Cinderella’s problems before disappearing from the story. 

The competing, palimpsestic Cinderella narratives embedded in *Cinderella is Dead* reflect the multivocal ways in which “fairytales register an effort on the part of both women and men to develop maps for coping with personal anxieties, family conflicts, social frictions and the myriad frustrations of everyday life” (Tatar 1999, p. xi). The resolution of the narrative with the death of both Amina and Manford, as well as Mersailles under Constance’s rule, opens into an uncertain future with perhaps more questions than answers, but one that also reflects what Zipes terms the “utopian spirit of the tales” (Zipes 2000, p. xix) though the exploration of what Sophia is searching for at the beginning of the novel—“alternative possibilities to life at ‘home’…for the miraculous transformation does not only involve the transformation of the protagonist but also the realization of a more ideal setting in which the hero-heroine can fulfil his or her potential” (Zipes 2000, p. xix). Sophia’s journey through Cinderella’s past to fix her present brings all the women of Mersailles to a new ‘home’, but this is only possible because of Amina’s actions. Conversely, it takes Amina’s interactions with Sophia and Constance to be willing to take action; she, as with Manford, has existed for over 200 years. Intergenerational dialogues are what spark change and awareness in her, having spent so long removed from the women of Mersailles and living alone in the White Wood. The three main female actors then demonstrate both “the appeal of fairytales as a map for lived experience and their limitations in providing actual guidance” (Williams 2021, p. 23). While comparing the different versions of the fairytale helps Sophia and Constance to understand what exactly they are facing, and eventually discover the truth of Manford’s rule, the real story of Cinderella ultimately does not help them to imagine a new future for their country, nor set them on any clear path. However, Amina’s characterization does serve to complicate the construction of older women, whether they might be fairy godmothers, wicked witches or simply older women who are mothers and mentors, as well as people in their own right. As such, *Cinderella is Dead* provides an interesting and provocative example of a fairytale narrative that upends constructions of age by allowing its older female characters an uncommon degree of agency and a map for readers that shows them that this agency is not something they must relinquish as they too inevitably age. 
